# Outcomes of Hospitalized Liver Cirrhosis Patients With COVID-19 Infection: A Retrospective Analysis

**DOI:** 10.7759/cureus.91524

**Published:** 2025-09-03

**Authors:** Ki Jung Lee, Parth Patel, Raffi Karagozian

**Affiliations:** 1 Internal Medicine, Tufts Medical Center, Boston, USA; 2 Internal Medicine, University of Cincinnati College of Medicine, Cincinnati, USA; 3 Gastroenterology and Hepatology, Tufts Medical Center, Boston, USA

**Keywords:** cirrhosis, covid-19, liver disease, malnutrition, mortality

## Abstract

Background

Patients with liver cirrhosis (LC) are at an increased risk of adverse outcomes associated with coronavirus disease 2019 (COVID-19). Existing studies have demonstrated a higher prevalence of malnutrition among COVID-19 patients. However, there is limited research assessing the impact of malnutrition on COVID-19 patients hospitalized with cirrhosis.

Methodology

We conducted a retrospective analysis of patients with LC admitted to hospitals in the United States in 2020 using the National Inpatient Sample (NIS) database. We compared in-hospital mortality, the risk for acute kidney injury (AKI), and length of stay (LOS) between malnourished and non-malnourished LC patients with COVID-19. Multivariable logistic regression analysis assessed the independent association between malnutrition in these patients and the outcomes of interest.

Results

Among 5,192 LC patients with COVID-19 and LC identified in the NIS database, 4,593 (88.5%) were not malnourished, and 599 (11.5%) were malnourished. The median age of non-malnourished patients was 63 (interquartile range (IQR) = 54-72) years, and that of malnourished patients was 64 (IQR = 56-72) years. Examining the baseline characteristics, the following did not have statistically significant differences: sex (male: non-malnourished: 60.4% vs. malnourished: 61.6%) and race (White: 50.5% vs. 49.9%). Malnourished patients with LC and COVID-19 were more likely to have hyponatremia (217; 36.2% vs. 1,200; 26.1%) and chronic kidney disease (CKD) (146; 24.4% vs. 928; 20.2%) but less likely to have hypertension (149; 24.9% vs. 1484; 32.3%), hyperlipidemia (141; 23.5% vs. 1441; 31.3%), obesity (75; 12.5% vs. 1010; 22.0%), and diabetes (53; 8.8% vs. 718; 15.6%). Malnourished patients had a significantly higher in-hospital mortality rate (171; 28.5%) compared to non-malnourished patients (836; 18.2%) (p < 0.001). Malnutrition in LC and COVID-19 patients was associated with an increased risk of in-hospital mortality (adjusted odds ratio (aOR) = 1.36, 95% confidence interval (CI) = 1.09-1.69, p < 0.01), AKI (aOR = 1.78, 95% CI = 1.47-2.16, p < 0.01), and LOS (unstandardized coefficient = 5.29, 95% CI = 4.52-6.06, p < 0.01).

Conclusions

Malnutrition in hospitalized LC patients with COVID-19 was associated with a higher risk of in-hospital mortality, AKI, and LOS. These findings highlight the importance of multidisciplinary management in addressing the nutritional status of COVID-19 patients with LC.

## Introduction

Liver cirrhosis (LC) is defined as an end-stage liver disease that is characterized by fibrotic tissue, resulting in disrupted liver architecture and impaired liver function. The scarring in cirrhosis leads to a loss of normal liver function, including the metabolism of toxins, production of proteins, notably albumin and clotting factors, and regulation of nutrients [1]. With pre-existing conditions, coronavirus disease 2019 (COVID-19) infection can be fatal. COVID-19 infection is a respiratory and systemic illness caused by the novel coronavirus SARS-CoV-2 [2]. It primarily infects cells via the angiotensin-converting enzyme receptor, which is abundant in the lungs, heart, kidneys, and other organs. The infection progresses to a hyperinflammatory response, known as a cytokine storm, resulting in tissue damage and systemic complications [3-5]. In patients with cirrhosis, this hyperinflammatory response can further exacerbate existing liver dysfunction and immune dysregulation, leading to decompensation and increased risk of adverse outcomes.

Malnutrition is a composite of clinical diagnosis categories, including protein-energy malnutrition, nutritional deficiencies of vitamins or minerals, muscle wasting, and other related conditions [6]. In clinical and research settings, malnutrition exhibits evidence of inadequate intake, absorption, or utilization of nutrients that lead to significant weight loss, muscle wasting, or biochemical markers for nutritional deficits [7]. Malnutrition is highly prevalent among COVID-19 patients with cirrhosis [8,9]. Patients with LC are at an increased risk of adverse outcomes when infected with COVID-19. This is associated with immune dysregulation that can exacerbate the severity of COVID-19 [9,10]. Therefore, we examined the outcomes of patients with LC and COVID-19. Despite the known prevalence of malnutrition and its impact on outcomes in both COVID-19 and cirrhosis, there is limited research specifically assessing the impact of malnutrition on COVID-19 patients hospitalized with cirrhosis. Therefore, our study also examines the impact of malnutrition and its impact on the various adverse outcomes in this specific population.

## Materials and methods

We conducted a retrospective cohort study using the Healthcare Cost and Utilization Project (HCUP) National Inpatient Sample (NIS) for 2020. The NIS, sponsored by the Agency for Healthcare Research and Quality, is a comprehensive database that collects discharge information from non-federal, non-rehabilitation, acute-care, and short-term hospitals. It represents approximately 20% of all hospital admissions and discharges in the United States and provides nationally representative estimates of patient and hospital characteristics. The data is de-identified and publicly available, so it does not require institutional review board approval.

Our study included patients admitted to hospitals between January and December 2020 with a primary diagnosis of LC. We used the International Classification of Diseases, Tenth Revision, Clinical Modification (ICD-10-CM) codes to identify discharge records with LC, and then stratified the data based on the presence or absence of COVID-19 infection. We collected patient comorbidities and relevant medical history from the discharge records using the ICD-10-CM codes [11].

The primary objective of our study was to investigate the impact of malnutrition on in-hospital mortality among LC patients with COVID-19. Secondary outcomes included factors associated with inpatient mortality as well as the influence of COVID-19 infection on the development of acute kidney injury (AKI) and hospital length of stay (LOS) among patients with LC.

We analyzed the patient data using SPSS version 27.0.1 (IBM Corp., Armonk, NY, USA), accounting for the complex sampling design of the NIS, which includes stratification, clustering, and weighting of patient and hospital-level data to ensure nationally representative results.

Continuous variables were presented as median and interquartile range (IQR), while categorical variables were presented as numbers and percentages. We used chi-square tests to compare proportions and t-tests to compare continuous variables. Multivariable logistic regression analyses were conducted, adjusting for potential confounders such as age, sex, race, median income, hospital bed size, hospital location, teaching status, insurance type, and comorbidities. We also conducted multivariate linear regression analysis to adjust for confounders in the secondary outcome of hospital LOS. The results were reported as odds ratios (ORs) and beta coefficients with 95% confidence intervals (CIs) as appropriate. Statistical significance was defined as a p-value <0.05 [12].

## Results

Of 6,471,165 total hospitalizations in the 2020 HCUP-NIS, 157,421 (2.4%) had a principal diagnosis of LC, including 5,197 (3.3%) with concomitant COVID-19 infection. Among LC patients with COVID-19, 599 (11.5%) were identified as malnourished (Table 1).

**Table 1:** Sociodemographic and clinical characteristics of liver cirrhosis patients with concomitant COVID-19. COPD = chronic obstructive pulmonary disease; CAD = coronary artery disease; CKD = chronic kidney disease; ERD = early renal disease; IQR = interquartile range

		Median (IQR)	Count	%
Age in years at admission		63 (54–72)	-	-
Sex	Male	-	3,145	60.5%
	Female	-	2,052	39.5%
Race (uniform)	White	-	2,560	50.5%
	Black	-	616	12.1%
	Hispanic	-	1,417	27.9%
	Asian or Pacific Islander	-	119	2.3%
	Native American	-	143	2.8%
	Other	-	219	4.3%
Comorbidities				
Hyponatremia	Yes	-	1,417	27.3%
Malnutrition	Yes	-	599	11.5%
Hypertension	Yes	-	1,633	31.4%
Hyperlipidemia	Yes	-	1,582	30.4%
Obesity	Yes	-	1,085	20.9%
COPD	Yes	-	420	8.1%
CAD	Yes	-	885	17.0%
CKD	Yes	-	1,074	20.7%
ERD	Yes	-	433	8.3%
Tobacco use	Yes	-	48	0.9%
Diabetes	Yes	-	771	14.8%
In-hospital complications				
Acute kidney injury	Yes	-	1,900	36.6%
Median household income national quartile for patient ZIP code	0–25th	-	1,943	38.5%
	26th–50th	-	1,296	25.7%
	51st–75th	-	1,021	20.2%
	76th–100th	-	785	15.6%
Primary expected payer (uniform)	Medicare	-	2,620	50.5%
	Medicaid	-	1,257	24.2%
	Private insurance	-	887	17.1%
	Self-pay	-	212	4.1%
	No charge	-	12	0.2%
	Other	-	198	3.8%
Hospital characteristics				
Region of the hospital	Northeast	-	923	17.8%
	Midwest	-	1,075	20.7%
	South	-	1,974	38.0%
	West	-	1,225	23.6%
Relative bed size category of hospital (strate)	Small	-	1,044	20.1%
	Medium	-	1,514	29.1%
	Large	-	2,639	50.8%
Location/teaching status of hospital (strata)	Rural	-	336	6.5%
	Urban nonteaching	-	854	16.4%
	Urban teaching	-	4,007	77.1%
Died during hospitalization	Yes	-	1,007	19.4%
Total		-	-	100.0%

The median age was 63 years (IQR = 54-72), and 3,145 (60.5%) were male. Racial distribution included 2,560 (50.5%) White, 1,417 (27.9%) Hispanic, 616 (12.1%) Black, 119 (2.3%) Asian or Pacific Islander, 143 (2.8%) Native American, and 219 (4.3%) Other. Common comorbidities included 1,417 (27.3%) hyponatremia, 599 (11.5%) malnutrition, 1,633 (31.4%) hypertension, 1,582 (30.4%) hyperlipidemia, 1,085 (20.9%) obesity, 420 (8.1%) chronic obstructive pulmonary disease, 885 (17.0%) coronary artery disease, 1,074 (20.7%) chronic kidney disease (CKD), 433 (8.3%) early renal disease (ERD), 48 (0.9%) tobacco use, and 771 (14.8%) diabetes. AKI occurred in 1,900 (36.6%) LC patients with COVID-19. The majority were insured through 2,620 (50.5%) Medicare and were admitted to 2,639 (50.8%) large and 4,007 (77.1%) urban teaching hospitals, with regional distribution highest at 1,974 (38.0%) in the South (Table 1).

When comparing malnourished and non-malnourished LC patients with COVID-19 (n = 599 vs. 4,593), baseline demographics were similar (age, sex, race, insurance, income, and hospital type). However, malnourished patients had higher rates of hyponatremia (217; 36.2% vs. 1,200; 26.1%), CKD (146; 24.4% vs. 928; 20.2%), and AKI (305; 50.9% vs. 1,595; 34.7%), but lower rates of hypertension (149; 24.9% vs. 1,484; 32.3%), hyperlipidemia (141; 23.5% vs. 1,441; 31.3%), obesity (75; 12.5% vs. 1,010; 22.0%), and diabetes (53; 8.8% vs. 718; 15.6%) (all p < 0.001). In-hospital mortality was significantly higher among malnourished patients (171; 28.5%) vs. non-malnourished patients (836; 18.2%, p < 0.001) (Table 2).

**Table 2:** Comparison of sociodemographic and clinical characteristics of malnourished and non-malnourished liver cirrhosis and COVID-19-positive patients. COPD = chronic obstructive pulmonary disease; CAD = coronary artery disease; CKD = chronic kidney disease; ERD = early renal disease; IQR = interquartile range

		Non-malnourished			Malnourished			P-value
		Median (IQR)	Count	Column %	Median (IQR)	Count	Column %	
Age in years at admission		63 (54–72)	-	-	64 (56–72)	-	-	0.128
Sex	Male	-	2,776	60.4%	-	369	61.6%	0.563
	Female	-	1,822	39.6%	-	230	38.4%	
Race (uniform)	White	-	2,269	50.5%	-	291	49.9%	0.200
	Black	-	531	11.8%	-	85	14.6%	
	Hispanic	-	1,271	28.3%	-	146	25.0%	
	Asian or Pacific Islander	-	101	2.2%	-	18	3.1%	
	Native American	-	124	2.8%	-	19	3.3%	
	Other	-	195	4.3%	-	24	4.1%	
Comorbidities								
Hyponatremia		-	1,200	26.1%	-	217	36.2%	<0.001
Hypertension		-	1,484	32.3%	-	149	24.9%	<0.001
Hyperlipidemia		-	1,441	31.3%	-	141	23.5%	<0.001
Obesity		-	1,010	22.0%	-	75	12.5%	<0.001
COPD		-	377	8.2%	-	43	7.2%	0.389
CAD		-	781	17.0%	-	104	17.4%	0.818
CKD		-	928	20.2%	-	146	24.4%	0.017
ERD		-	391	8.5%	-	42	7.0%	0.214
Tobacco use		-	44	1.0%	-	4	0.7%	0.487
Diabetes		-	718	15.6%	-	53	8.8%	<0.001
In-hospital complications								
Acute kidney injury	Yes	-	1,595	34.7%	-	305	50.9%	<0.001
Median household income national quartile for patient ZIP Code	0–25th	-	1,725	38.7%	-	218	37.4%	0.815
	26th–50th	-	1,137	25.5%	-	159	27.3%	
	51st–75th	-	906	20.3%	-	115	19.7%	
	76th–100th	-	694	15.6%	-	91	15.6%	
Primary expected payer (uniform)	Medicare	-	2,302	50.2%	-	318	53.1%	0.102
	Medicaid	-	1,107	24.1%	-	150	25.0%	
	Private Insurance	-	787	17.2%	-	100	16.7%	
	Self-Pay	-	195	4.3%	-	17	2.8%	
	No charge		12	0.3%		0	0.0%	
	Other	-	184	4.0%	-	14	2.3%	
Hospital characteristics								
Region of the hospital	Northeast	-	834	18.1%	-	89	14.9%	0.008
	Midwest	-	924	20.1%	-	151	25.2%	
	South	-	1,764	38.4%	-	210	35.1%	
	West	-	1,076	23.4%	-	149	24.9%	
Relative bed size category of hospital (strata)	Small	-	930	20.2%	-	114	19.0%	0.371
	Medium	-	1,325	28.8%	-	189	31.6%	
	Large	-	2,343	51.0%	-	296	49.4%	
Location/Teaching status of hospital (strata)	Rural	-	309	6.7%	-	27	4.5%	0.117
	Urban nonteaching	-	754	16.4%	-	100	16.7%	
	Urban teaching	-	3,535	76.9%	-	472	78.8%	
Died during hospitalization	Yes	-	836	18.2%	-	171	28.5%	<0.001
Total		-	4,593	100.0%	-	599	100.0%	-

Multivariable logistic regression (Table 3) identified older age (adjusted OR (aOR) = 1.031, 95% CI = 1.023-1.039), Hispanic race (AOR = 1.303), malnutrition (AOR = 1.359), and AKI (AOR = 5.577) as independent predictors of mortality (all p < 0.01). Conversely, hypertension, tobacco use, and diabetes were associated with lower odds of death.

**Table 3:** Multivariable logistic regression of factors associated with inpatient mortality among hospitalized patients with liver cirrhosis and COVID-19. Variable(s) entered in step 1: age in years at admission, indicator of sex, race (uniform), hyponatremia, malnutrition, hypertension, hyperlipidemia, obesity, COPD, CAD, CKD, ERD, tobacco use, diabetes, acute kidney injury, median household income national quartile for patient ZIP Code, primary expected payer (uniform), region of the hospital, relative bed size category of hospital (strata), and location/teaching status of hospital (strata). COPD = chronic obstructive pulmonary disease; CAD = coronary artery disease; CKD = chronic kidney disease; ERD = early renal disease; aOR = adjusted odds ratio; CI = confidence interval

		P-value	aOR	95% CI for AOR	
				Lower	Upper
Age in years at admission		0.000	1.031	1.023	1.039
Female		0.946	0.995	0.847	1.167
Race	White	0.006	-	-	-
	Black	0.739	0.958	0.745	1.232
	Hispanic	0.009	1.303	1.069	1.589
	Asian or Pacific Islander	0.637	1.129	0.682	1.869
	Native American	0.215	1.386	.827	2.321
	Other	0.001	1.783	1.248	2.548
Comorbidities					
Hyponatremia		0.925	1.008	0.850	1.195
Malnutrition		0.006	1.359	1.093	1.689
Hypertension		0.000	0.686	0.562	0.837
Hyperlipidemia		0.189	0.886	0.740	1.061
Obesity		0.571	1.058	0.870	1.287
COPD		0.123	0.786	0.579	1.067
CAD		0.850	0.980	0.793	1.211
CKD		0.000	0.653	0.529	0.806
ERD		0.040	1.362	1.014	1.830
Tobacco use		0.025	0.188	0.043	0.813
Diabetes		0.011	0.712	0.548	0.926
In-hospital complications					
Acute kidney injury		0.000	5.577	4.708	6.606
Median household income national quartile for patient ZIP Code	0-25th	0.286	-	-	-
	26th-50th	0.956	0.994	0.817	1.210
	51st-75th	0.071	0.815	0.653	1.018
	76th-100th	0.720	0.956	0.749	1.221
Primary expected payer (uniform)	Medicare	0.654	-	-	-
	Medicaid	0.313	1.127	0.894	1.420
	Private Insurance	0.400	0.901	0.708	1.148
	Self-Pay	0.814	1.054	0.678	1.639
	No charge	0.637	0.600	0.072	5.002
	Other	0.581	1.129	0.734	1.737
Region of the hospital	Northeast	0.108	-	-	-
	Midwest	0.038	0.766	0.595	0.986
	South	0.045	0.795	0.636	0.995
	West	0.038	0.770	0.601	0.986
Relative bed size category of hospital (strata)	Small	0.227	-	-	-
	Medium	0.090	1.214	0.970	1.520
	Large	0.364	1.102	0.894	1.358
Location/Teaching status of hospital (strata)	Rural	0.517	-	-	-
	Urban nonteaching	0.438	1.165	0.792	1.715
	Urban teaching	0.846	1.035	0.729	1.471
Constant		0.000	0.018	-	-

Predictors of AKI (Table 4) included older age, male sex, Black race, hyponatremia, malnutrition, CKD, and end-stage renal disease (ESRD). Diabetes was associated with a lower risk.

**Table 4:** Adjusted odds ratio for acute kidney injury among hospitalized liver cirrhosis patients with concomitant COVID-19. Variable(s) entered in step 1: age in years at admission, indicator of sex, race (uniform), hyponatremia, malnutrition, hypertension, hyperlipidemia, obesity, COPD, CAD, CKD, ERD, tobacco use, diabetes, acute kidney injury, median household income national quartile for patient ZIP Code, primary expected payer (uniform), region of the hospital, relative bed size category of hospital (strata), and location/teaching status of hospital (strata). COPD = chronic obstructive pulmonary disease; CAD = coronary artery disease; CKD = chronic kidney disease; ERD = early renal disease; aOR = adjusted odds ratio; CI = confidence interval

		P-value	aOR	95% CI for AOR	
				Lower	Upper
Age in years at admission		0.000	1.013	1.007	1.020
Females		0.028	0.861	0.753	0.984
Race	White	0.031	-	-	-
	Black	0.002	1.385	1.125	1.706
	Hispanic	0.687	0.967	0.819	1.141
	Asian or Pacific Islander	0.821	0.952	0.620	1.460
	Native American	0.416	1.189	0.783	1.805
	Other	0.368	1.159	0.841	1.597
Comorbidities					
Hyponatremia		0.000	1.651	1.433	1.902
Malnutrition		0.000	1.779	1.468	2.157
Hypertension		0.137	0.889	0.762	1.038
Hyperlipidemia		0.587	0.959	0.824	1.116
Obesity		0.450	0.939	0.799	1.105
COPD		0.002	0.676	0.527	0.867
CAD		0.838	1.019	0.848	1.224
CKD		0.000	4.624	3.874	5.518
ERD		0.000	0.367	0.275	0.491
Tobacco use		0.157	1.652	0.824	3.311
Diabetes		0.000	0.591	0.483	0.724
Median household income national quartile for patient ZIP Code	0–25th	0.019	-	-	-
	26th–50th	0.073	1.162	0.986	1.369
	51st–75th	0.690	1.038	0.866	1.244
	76th–100th	0.085	0.835	0.681	1.025
Primary expected payer (uniform)	Medicare	0.094	-	-	-
	Medicaid	0.202	1.132	0.936	1.369
	Private insurance	0.092	1.182	0.973	1.436
	Self-pay	0.282	0.823	0.577	1.174
	No charge	0.098	0.162	0.019	1.395
	Other	0.328	1.187	0.842	1.676
Region of the hospital	Northeast	0.809	-	-	-
	Midwest	0.343	0.903	0.731	1.115
	South	0.437	0.927	0.767	1.121
	West	0.543	0.937	0.761	1.154
Relative bed size category of hospital (strata)	Small	0.290	-	-	-
	Medium	0.148	1.147	0.952	1.381
	Large	0.157	1.132	0.953	1.344
Location/Teaching status of hospital (strata)	Rural	0.019	-	-	-
	Urban nonteaching	0.335	1.169	0.851	1.606
	Urban teaching	0.023	1.394	1.048	1.855
Constant		0.000	0.124	-	-

Linear regression (Table 5) showed that malnutrition, hyponatremia, obesity, ESRD, and AKI were significantly associated with increased hospital LOS, with AKI having the strongest effect (B = 3.589, p < 0.001).

**Table 5:** Multivariate linear regression of length of stay among hospitalized liver cirrhosis patients with concomitant COVID-19. Dependent variable: length of stay. Predictors (constant): location/teaching status of hospital (strata), obesity, tobacco use, CAD, diabetes, hyponatremia, relative bed size category of the hospital (strata), malnutrition, median household income national quartile for patient ZIP code, indicator of sex, COPD, region of the hospital, ERD, primary expected payer (uniform), acute kidney injury, race (uniform), hypertension, hyperlipidemia, age in years at admission, and CKD. COPD = chronic obstructive pulmonary disease; CAD = coronary artery disease; CKD = chronic kidney disease; ERD = early renal disease; CI = confidence interval

	Unstandardized coefficients (B)	P-value	95% CI for B	
			Lower bound	Upper bound
(Constant)	3.623	0.003	1.271	5.974
Sociodemographic characteristics				
Age in years at admission	-0.004	0.739	-0.026	0.018
Females	-0.590	0.023	-1.100	-0.080
Comorbidities				
Hyponatremia	2.713	0.000	2.156	3.269
Malnutrition	5.291	0.000	4.519	6.064
Hypertension	-0.205	0.499	-0.800	0.390
Hyperlipidemia	0.010	0.974	-0.564	0.584
Obesity	1.054	0.001	0.444	1.665
COPD	-0.809	0.081	-1.720	0.101
CAD	-0.251	0.481	-0.947	0.446
CKD	-0.873	0.017	-1.593	-0.154
ERD	1.306	0.007	0.354	2.258
Tobacco use	-0.603	0.663	-3.321	2.114
Diabetes	-0.457	0.216	-1.181	0.267
In-hospital complications				
Acute kidney injury	3.589	0.000	3.039	4.139

## Discussion

In this retrospective cohort study, the impact of malnutrition on those hospitalized with COVID-19 and cirrhosis was investigated. After stratifying, clustering, and weighting, those with malnutrition were found to have significantly higher AKI, in-hospital mortality, and total LOS. Similar findings have been reported in other studies, demonstrating malnutrition as an independent risk factor for greater inpatient mortality and LOS [13]. Specifically, with the COVID-19 pandemic, prior studies have indicated that malnutrition is associated with increased mortality in those diagnosed with COVID-19 who required inpatient admission [14,15]. Independently, cirrhosis is associated with increased mortality risk when combined with COVID-19 compared to COVID-19 alone [16]. Therefore, the present analysis highlights that malnutrition is a notable risk factor for those who already have significant comorbidity of cirrhosis and need hospitalization for COVID-19.

The association of AKI and malnutrition in critical illness states has been demonstrated in previous studies [17,18]. Our study is consistent with others in which patients with COVID-19, malnutrition was prevalent and exacerbated the risk of AKI [19]. Especially those hospitalized with COVID-19, patients experienced multifactorial changes, triggered by direct cellular infiltration via the angiotensin-converting enzyme 2 receptor pathway utilized by the SARS-COV-2 virus [20,21]. More importantly, with an increased inflammatory state, catabolism is enhanced and anabolism is suppressed, which leads to hypoxia and hypoperfusion, further aggravated by the malnourished states with lower reserve [18,22-25]. Consequently, our study showed that AKI is a strong predictor for increased in-hospital mortality, consistent with previous studies [24,26]. As delineated above, a combination of systemic inflammation, hypoperfusion, and AKI leads to fluid and electrolyte imbalances, acid-base disturbances, and a compromised immune response that worsens the severity of the underlying critical illness and increases mortality [19,27,28]. In COVID-19 and cirrhosis patients, AKI is associated with direct viral invasion of renal cells, decreased effective arterial blood volume, and systemic vasoconstriction that compounds the effects of liver and kidney dysfunction [29,30].

Malnutrition is prevalent in both cirrhotic and critically ill COVID-19 patients due to reduced nutrient intake, altered metabolism, and chronic disease burden [29,30]. In cirrhosis, poor nutritional status is linked to sarcopenia and hypoalbuminemia, which aggravates systemic inflammation and impacts the body’s ability to manage infections and recover from AKI. Similarly, in COVID-19, malnutrition accelerates disease progression by worsening immune suppression, increasing susceptibility to infections, and delaying recovery [31]. With heightened severity, the subsequent in-hospital mortality is raised as well. Evidence indicates that malnutrition at the population level is associated with higher mortality rates from COVID-19. Thomas et al. explored the metabolic impact of COVID-19, which showed that serum metabolites in infected patients had higher levels of circulating free fatty acids, particularly in individuals with high inflammatory cytokine levels [14,15]. Furthermore, significant disruptions in nitrogen and carbon metabolism are likely linked to the prolonged and progressive hypermetabolism observed in COVID patients [32,33]. The severe inflammatory response, or cytokine storm, triggered by the host’s immune system during infection could have been implicated in the loss of skeletal muscle that can be seen in respiratory illness [34,35].

Adequate nutrition is necessary for the immune system to function appropriately. Malnutrition has a profound impact on clinical outcomes. Several factors, as discussed above, heighten vulnerability to complications such as infections, hepatic decompensation, and AKI, which extend hospitalization duration [36,37]. According to studies using the Global Leadership Initiative on Malnutrition criteria, malnutrition in cirrhosis patients correlates strongly with prolonged hospital stays and increased in-hospital mortality [38,39]. Studies have shown that malnourished COVID-19 patients face a higher risk of delirium, prolonged mechanical ventilation, and secondary infections that compound the clinical course. The combination of COVID-19 and pre-existing conditions such as cirrhosis amplifies these risks as both diseases share mechanisms of systemic inflammation, immune suppression, and nutritional depletion [36,37]. These result in prolonged hospitalizations.

Interestingly, our study showed a similar trend in the opposite spectrum of patients. Obesity was related to increased length of hospitalization. Obesity contributes to a chronic low-grade inflammatory state, which worsens when COVID-19 infection causes hyperinflammation [40]. This leads to higher rates of infection, respiratory failure, acute respiratory distress syndrome, and longer intensive care unit stay. Both malnutrition and obesity in cirrhosis patients are independent predictors of adverse clinical outcomes, including prolonged hospital stays [41-43]. Obesity, defined by body mass index (BMI), does not necessarily mean that a patient does not have sarcopenia, as BMI can be falsely elevated with ascites [44,45].

Our study has notable findings with a large national database that emphasize malnutrition is a risk factor for those with cirrhosis who are infected with COVID-19. With the increased prevalence of AKI, in-hospital mortality, and prolonged hospitalization, these populations highlight the critical need for timely nutritional assessment and intervention. Those with cirrhosis are affected at multiple levels and organs, making a multidisciplinary approach crucial [46,47]. One of the most important aspects of management is nutritional support. This should be tailored to address hypermetabolism, inflammation, and protein-energy deficit [48,49]. Consequently, they can mitigate muscle wasting and immune dysfunction, thereby improving recovery rates and leading to shortened hospitalizations [50-52]. The lasting impact would be meaningful to both patients and hospitals.

This study has a few limitations. The diagnoses were limited based on the filled ICD codes. Given that they were coded by an individual, individual variations could have persisted. The NIS database does not include specific laboratory values, including albumin, kidney function, or Model for End-Stage Liver Disease score, to assess the severity of liver disease. Finally, as this is a retrospective study, causality cannot be established.

## Conclusions

The impact of malnutrition in cirrhosis patients infected with COVID-19 included higher rates of AKI, mortality, and longer hospitalizations. Malnutrition is a dynamic process that puts patients at risk of developing a variety of adverse effects. Addressing inadequate nutrition plays a pronounced role in enhancing the immune system, decreasing the risk of infections and hepatic complications, and decreasing the length of hospitalization for the critically ill.
